# Supplementary material for: Measuring Rural Food Environments for Local Action in Australia: A Systematic Critical Synthesis Review
Source: Int J Environ Res Public Health. 2019 Jul 7;16(13):2416. doi: 10.3390/ijerph16132416 (PMC6651399; doi:10.3390/ijerph16132416)
Supplement: Supplementary file 1 [file ijerph-16-02416-s001.pdf]

Supplementary File S1: Data Extraction Table – Quality and Characteristics of Tools

Author	Geographical Setting	Consumer or Community Food Environment	Attribute/s Measured	Tool/s Used	Detail/s of Measure	Psychometric Characteristics of Tool/s	Context
**Cuttler et al. (2018)[34]**	Rural; Western Victoria, Australia	Consumer	Price	Victorian Healthy Food Basket (VHFB)	Stores identified through local government food outlet databases; data collected once a year for 3 consecutive years; followed VHFB protocol; also collected store type; distance from largest urban centre in area; SEIFA	Not specifically mentioned in article	45 stores (2014, 2015); 48 stores (2016) across 5 rural local governments in Western Victoria (Greater Geelong, Colac Otway, Golden Plains, Surf Coast and Queenscliff)
**Love et al. (2018)[35]**	Rural; 10 towns in a rural local government, Victoria, Australia	Consumer	Price; Price differential; Affordability	Healthy Diets Australian Standardised Affordability and Price (ASAP) tool	Food outlets identified using business listings, then ground truthing; all operating at time of survey included; data collected in pairs over 1 week	Followed ASAP protocol; Validity testing	40 food outlets across 10 towns within 1 rural local government in Victoria
**Whelan et al. (2018)[36]**	Rural; 10 towns in a rural local government, Victoria, Australia	Consumer	Available healthy options; Quality; Price	Nutrition Environment Measurement Survey - Stores (NEMS-S) / Restaurants (NEMS-R) (modified for Australian context)	Food outlets identified using business listings, then ground truthing; 2 service stations excluded, with all others operating at time included; data collected in pairs	Followed NEMS-S and NEMS-R protocols; undertook NEMS training; pilot tested for face validity with modifications for rural Australian context; data collected in pairs; high degree of test-re-test reliability	Census audit using ground truthing across 10 towns within 1 rural local government in Victoria; 11 food stores (5 supermarkets, 6 general stores); 27 food service outlets (no fast food chains - takeaways, bakeries, pubs, cafes)
**DuBreck et al. (2018)[52]**	Rural; Middlesex County, Southwestern Ontario, Canada	Consumer & Community	Type and Location of Outlets; Accessibility; Available Healthy Options; Promotion; Placement; Price; Nutrition Information	CMA (Childrens Menu Assessment tool) - an extension of NEMS-R for children's menus	CMA extends to whole grains, fruits, vegetables, other non-fried items, nutritional information, toy promotions, and branded marketing; also collected neighbourhood level socioeconomic distress index - educational attainment; income; employment rate; single parent families	High degree of inter-rater reliability	1071 restaurants, with 364 identified as having separate children's menus; 174 unique children's menus
**Larson et al. (2017)[44]**	Rural; Nicollet County, Minnesota, USA	Consumer	Available healthy options; Quality; Price	Minnesota Food Pilot 2016: Corner/ Convenience Store Assessment, Minnesota Department of Health (MDH)	Auditors trained and worked in pairs; 49-question audit (provision of specific foods, quality, price	Not specifically mentioned in article	Store outlet information provided by State Health Improvement Program (SHIP); 22 of 24 stores audited (15 gas

Author	Geographical Setting	Consumer or Community Food Environment	Attribute/s Measured	Tool/s Used	Detail/s of Measure	Psychometric Characteristics of Tool/s	Context
					and quantity present); also unhealthy and/or healthy foods available near checkout		stations; 3 discount stores; 2 drug stores; ethnic market; meat market)
**Palermo et al. (2016)**[37]	Rural and Urban; 26 local governments in Victoria, Australia	Consumer	Price	Victorian Healthy Food Basket (VHFB)	44 'healthy' and 10 'discretionary' food and drink items for a family of 4 for a fortnight	Not specifically mentioned in article	Stratified random sample of 115 stores from 26 local governments
**Byker Shanks et al. (2015)**[45]	Rural; Montana, USA	Consumer	Available healthy options; Quality; Price	Nutrition Environment Measurement Survey - Stores (NEMS-S)	Followed NEMS-S protocol	Not specifically mentioned in article	Random selection of rural counties; 20 stores in 17 towns in 12 counties; 17 rural stores vs 3 urban stores in urban control county
**Byker Shanks, Jilcott Pitts & Gustafson (2015)**[46]	Rural and Urban; Kentucky, North Carolina and Montana, USA	Consumer & Community	Accessibility; Available Healthy Options; Quality	Farmers' Market Audit Tool [F-MAT]	Based on the NEMS-S tool	Reviewed face validity of tool with content experts; Inter-rater reliability high across all sites but one; Discriminant validity also assessed as good	6 counties across 3 states, one rural and one urban county in each; one farmers' market in each county assessed
**Chapman et al. (2014)**[38]	Rural and Urban; New South Wales, Australia	Consumer	Price	Queensland Healthy Food Basket Survey (QLD HFAB);<br><br>Fruit and Vegetable Variety Score	44 Item healthy food basket; contains core food groups described in the 2003 Dietary Guidelines;<br>Variety Score calculated by summing all the numbers of varieties of the 30 different fresh fruit and vegetables	Inter-rater reliability assessed	2006 = 149 stores<br>2008 = 105 stores<br>2009 = 129 stores<br>Convenience sampled
**Pereira et al. (2014)**[32]	Rural; New Ulm, Minnesota, USA	Consumer	Available Healthy Options; Quality; Price; Nutrition Information; Promotion	Nutrition Environment Measurement Survey - Stores (NEMS-S) / Restaurants (NEMS-R)	NEMS-S: Score of up to 66; NEMS-R: Score of up to 90; For both measures a higher score represents conditions more conducive to healthy eating	Tools selected because they were the "best available" at the time of the research based on psychometric properties	34 Restaurants<br>3 Grocery Stores<br>5 Convenience Stores
**Pollard et al. (2014)**[22]	Rural and Urban; Western Australia	Consumer	Price; Quality	Queensland Healthy Food Basket Survey (QLD HFAB); Quality Tool based on NEMS-S	Followed protocols for QLD HFAB methodology and NEMS-S	Not discussed	144 Supermarket/ Grocery stores in WA; Stratified random sample based on ARIA+ category and SES
**Tseng et al. (2014)**[39]	Rural and Urban; Victoria, Australia	Community	Type and Location of Outlets	Development of Obesogenicity Index	Index based on 9 items; 3 items from each of 3 domains with food resources one domain, (number of chain supermarkets, green grocers and fast food restaurants	Pilot index; limitations and areas to improve discussed	Used a geographic buffer of 2km [road network distance] to represent an accessible area around a participant's home.

Author	Geographical Setting	Consumer or Community Food Environment	Attribute/s Measured	Tool/s Used	Detail/s of Measure	Psychometric Characteristics of Tool/s	Context
					within 2km buffer of participants home)		
**Pitts et al. (2013)[33]**	Rural and Urban; Northern Carolina, USA	Consumer	Available Healthy Options; Price; Quality	Nutrition Environment Measurement Survey - Stores - Revised (NEMS-S-Rev)	According to NEMS-S-Rev protocol the overall score summing availability, pricing and quality scores ranged from -12 to 64	Inter-rater reliability of >80% achieved for all food store audits in study	42 Food Stores<br>33 Corner stores<br>9 Chain supermarkets
**^Innes-Hughes et al. (2012)[40]**	Rural;<br>3 rural towns in Southern New South Wales, Australia	Consumer & Community	Available Healthy Options;<br><br>Type and Location of Outlets	Measured proximity using own formula;<br>Measured in-store availability using two checklists developed for the research;<br>Outlets classified according to system developed for the research	2 Checklists developed to record availability of healthy and unhealthy foods in food retail and food service outlets based on modification of NEMS-S;<br>GPS used to plot location of outlets	Checklists had excellent inter-rater and test-retest reliability;<br>Direct observation of food environment had face and content validity as well as good criterion validity	Temora = 28 outlets;<br>Ariah Park = 2 outlets;<br>Narrandera = 28 outlets;<br>Hay = 19 outlets
**Ward et al. (2012)[41]**	Rural and Urban*<br>(*separate study);<br>10 rural towns in South Australia;<br>Results compared to metropolitan data collected in a separate study	Consumer	Price	Victorian Healthy Food Basket (VHFB)	Followed protocol for VHFB	Not specifically mentioned in article	144 Supermarkets across 10 towns
**Sadler, Gilliland & Arku (2011)[53]**	Rural;<br>Middlesex County, Ontario, Canada	Community	Type and Location of Outlet	GIS Mapping	Store categories based on Health Inspector Database	Not discussed	Calculated distance by road from all residences in rural country of Middlesex to the nearest of each of the types of food outlet
**Sharkey et al. (2011)[47]**	Rural;<br>6 rural counties in Central Texas, Brazos Valley region, USA	Consumer & Community	Type and Location of Outlet;<br><br>Available Healthy Options;<br>Promotion;<br>Nutrition Information	Two part observational tool developed.	Part 1: All store types observed, coded and GIS mapped<br><br>Part 2: Assessment of the menu items including: availability of healthier options, identification of nutritional information and preparation methods	Not specifically mentioned in article	261 Retail food outlets surveyed;<br>205 fast-food opportunities identified: 84 fast food outlets (41%); 12 traditional food stores (5.8%); 109 convenience stores (53.2%)
**Smith et al. (2010)[50]**	Rural and Urban;<br>Island, Rural, Small Town and Urban Scotland	Consumer & Community	Type and location of outlet;<br><br>Available Healthy Options	GIS Mapping and measures based on network travel times;<br><br>Healthy Eating Indicator Shopping Basket (HEISB) Tool	Calculated travel time to nearest outlet from residential centroids;<br><br>Study used availability of 12 fresh fruit and vegetable	Refers to HEISB methodology but does not specifically consider psychometric characteristics of tools utilised.	9 randomly selected sentinel sites representing different urban-rural and SES classifications<br><br>457 Food retail facilities

Author	Geographical Setting	Consumer or Community Food Environment	Attribute/s Measured	Tool/s Used	Detail/s of Measure	Psychometric Characteristics of Tool/s	Context
					items of the 17 listed in the HEISB		
**Wang et al. (2010)[54]**	Rural and Urban; Waikato/Lakes areas, New Zealand	Consumer	Available Healthy Options; Price	No existing tool mentioned	Availability of 15 healthy items, 8 food categories for price comparison between regular and 'healthy' alternatives; availability of 'healthy' choices in takeaway outlets	Not discussed	1230 Food outlets (n=473 urban) (n= 757 rural)
**Cummins et al. (2009)[51]**	Rural and Urban; Island, Rural, Small Town and Urban Scotland	Consumer	Quality	Quality section of the Healthy Eating Indicator Shopping Basket (HEISB) tool	12 fresh fruit and vegetable items rated on a Likert scale for quality: 1 = poor, 2 = medium and 3 = good using specific criteria	Refers to HEISB methodology ; Did not examine inter-rater reliability (noted in article)	288 Food stores in 10 communities; communities selected to reflect a range of urban-rural settings
**Palermo et al. (2008)[42]**	Rural; 18 towns in 14 local governments in Victoria, Australia	Consumer	Price	Victorian Healthy Food Basket [VHFB]	44 core foods selected from the Australian Guide to Healthy Eating and two 'extra foods' (soft drink and a chocolate bar)	Not discussed	Convenience sample 34 Supermarkets in rural and regional areas<br><br>Population of towns varied from <3000 to 80,000
**Hosler et al. (2008)[48]**	Rural and Urban; Albany, Columbia and Greene counties in New York State, USA	Consumer & Community	Type & Location Accessibility;<br><br>Available Healthy Options	Developed a survey tool, store classification scheme and method to calculate store density as a measure of fresh fruit and vegetable availability	Fruit and vegetable store density per 10,000 residents; Check list for availability of fruits and vegetables; Collected information re: number of registers and store hours	Inter-rater reliability tested	263 Retail stores and farmers markets
**Creel et al. (2008)[49]**	Rural; 6 counties in rural Texas, USA	Consumer & Community	Promotion; Available Healthy Options; Nutrition Information;<br><br>Type & Location of Food Outlet; Accessibility	Two-part observational survey instrument developed based on prior restaurant audits, the 2005 Dietary Guidelines for Americans and input from local registered Dietitians	Part 1: Store type, store hours, store exterior, condition of parking lot, promotions of fast food or healthy food, store interior and store size (number of tables or cash registers)<br><br>Part 2: Assessment of menu items: availability of healthier options, identification of nutritional information and preparation methods	Not examined (Authors identify this as a study limitation)	84 Fast-Food outlets; 109 Convenience stores; 12 Supermarkets/Grocery stores
**Herzfeld & McManus (2007)[43]**	Rural and Urban Fringe; Tasmania, Australia	Consumer	Available Healthy Options; Quality; Price	Pilot tool developed	Availability measured based on surveyed product being in store and quantities available (insufficient, moderate or sufficient);	Inter-rater reliability examined and determined as poor; No validity testing	Tool piloted in 4 independent stores in 2 communities

Author	Geographical Setting	Consumer or Community Food Environment	Attribute/s Measured	Tool/s Used	Detail/s of Measure	Psychometric Characteristics of Tool/s	Context
					Quality measured across 15 categories of vegetables and 9 categories of fruit;<br>Price per kilogram		
**Martínez-Donate et al. (2015)[31]**	Rural;<br>2 Mid-Western USA rural communities (matched control and intervention sites) 30 miles apart	Consumer	Available Healthy Options;<br>Promotion;<br>Price;<br>Nutrition Information;<br>Quality	Nutrition Environment Measurement Survey - Stores (NEMS-S) / Restaurants (NEMS-R)	Protocol for NEMS-S and NEMS-R	NEMS –S & NEMS-R previously validated, high degrees of inter-rater and test-retest reliability and good validity	Matched intervention (6000 population) and control (26,000 population) towns<br><br>Intervention site:<br>7 Restaurants (6 locally owned, sit down restaurants; and 1 franchise chain fast casual restaurant);<br>2 Supermarkets (both chain grocery stores)

Supplementary File S2: Critical Review of Literature – Appraisal Summary and Narrative Analysis (^indicates Australian studies)

Author/Year	**Critical Appraisal Summary** (Numbers 1 to 22 in row 1 relate to the STROBE Checklist and TREND Statement items)																						
	**1**	**2**	**3**	**4**	**5**	**6**	**7**	**8**	**9**	**10**	**11**	**12**	**13**	**14**	**15**	**16**	**17**	**18**	**19**	**20**	**21**	**22**	**Total**
**^Cuttler et al. (2018)[34]**									X										X		X	X	18/22
**^Love et al. (2018)[35]**																							22/22
**^Whelan et al. (2018)[36]**																							22/22
**DuBreck et al. (2018)[52]**																	n/a				n/a		20/20
**Larson et al. (2017)[44]**									X		X	X					X			X	X		16/22
**^Palermo et al. (2016)[37]**									X			X			X				X		X		17/22
**Byker Shanks et al. (2015)[45]**									X	X			n/a	n/a			X				X		16/20
**Byker Shanks, Jilcott Pitts & Gustafson (2015)[46]**										X			n/a	n/a	n/a	n/a						X	16/18
**^Chapman et al. (2014)[38]**							X		X	X		X	X			X			X				15/22
**Pereira et al. (2014)[32]**									X		X	X			X	n/a	n/a						16/20
**^Pollard et al. (2014)[22]**							X												X				20/22
**^Tseng et al. (2014)[39]**	X								X										X				19/22
**Pitts et al. (2013)[33]**												n/a		X						X	X		18/21
**^Innes-Hughes et al. (2012)[40]**							X					n/a					n/a		X			X	17/20
**^Ward et al. (2012)[41]**	X						X			X			X								X	X	16/22
**Sadler, Gilliland & Arku (2011)[53]**									n/a			X		X				X	X				17/21
**Sharkey et al. (2011)[47]**						X	n/a		n/a	X			n/a	X									16/19
**Smith et al. (2010)[50]**								X					X	X									19/22
**Wang et al. (2010)[54]**				X			n/a		n/a	X			X	X					X	X			14/20
**Cummins et al. (2009)[51]**							n/a		n/a				X	X									18/20
**^Palermo et al. (2008)[42]**							X		X	X			X									X	17/22
**Hosler et al. (2008)[48]**							X		n/a					X	X		n/a				X		16/20
**Creel et al. (2008)[49]**							n/a		n/a	X			X	X		n/a	n/a	X					14/18
**^Herzfeld & McManus (2007)[43]**				X	X		X	X	X			X	X	X		n/a	n/a					X	11/20
**Martínez-Donate et al. (2015)[31]**				X	X			X		X	X		X	X	X	n/a	n/a	n/a	X				10/19

Author/s	Study Aim/s	Rurality defined	Food/Nutrition Environment conceptual framework	Study type	Findings	Comments
**^Cuttler et al. (2018)[34]**	To measure the cost of healthy foods across a geographically defined region and examine factors related to changing food prices to assist in health promotion practices	No	No	Longitudinal (2014, 2015, 2016); Observational	Cost of the healthy food basket was the highest in stores >15 km from the major regional centre and in areas with only one supermarket. All food groups except fruit and vegetables decreased in price between 2014 and 2016.	**Strengths:**<br>• Validated VHFB tool<br>**Limitations**:<br>• No ground truthing mentioned<br>• Changes in store numbers over time;<br>• Eligibility of some stores limited due to unavailable VHFB items (does not state number of stores excluded)<br>**Recommendations:**<br>• This project highlights distance from regional centres and competition as possible factors that influence the cost of healthy food. This is valuable insight for health promoters in designing both local level interventions aimed at improving access to healthy food and influencing regional food systems
**^Love et al. (2018)[35]**	To assess price, price differential and affordability of the recommended (healthy) and current diets in a rural local government in Victoria, Australia	Yes<br>Guide to Remoteness Classifications, Australian Institute of Health and Welfare	Yes,<br>Glanz et al 2005	Cross-sectional; Observational	Across the local government, the recommended diet was cheaper than the current diet, costing an average 81.1% of the current diet budget. Across the local government, the recommended diet would expend 30–32%, and the current diet would expend 37–40%, of a median and low-income household.	**Strengths:**<br>• Adds to the ASAP studies for a rural context;<br>• Used ground truthing and census of stores, rather than small random sample;<br>• All data collectors were trained using the ASAP protocol<br>**Limitations:**<br>• Sample size small given rural geography which limited statistical analysis;<br>• 'Outshopping' not accounted for;<br>• ASAP tool needs adaption for rural areas (different brands, missing products; different reference households to reflect community ie: single elderly; couple no children)<br>**Recommendations:**<br>• Establishment of a reliable demand-supply cycle that would be economically viable for small food retailers<br>• Shaping appropriate interventions at the individual, organizational, community and policy level
**^Whelan et al. (2018)[36]**	To describe the food environment in a rural Australian community to inform the development of community-relevant food supply interventions	Yes<br>Guide to Remoteness Classifications, Australian Institute of Health and Welfare	Yes,<br>Glanz et al 2005	Cross-sectional; Observational	Food stores scored poorly on food availability and comparative pricing; healthier options were more expensive than their unhealthy alternative; and variable quality of fresh fruit and vegetables. The availability of food service outlets was more predominant than food stores, with the majority receiving low scores indicative of healthy choices being generally difficult to obtain across the local government.	**Strengths:**<br>• Validated NEMS tools used;<br>• First study to apply these tools to Australian rural context;<br>• Census audit, therefore representative of the local government area;<br>• All data collectors were trained using the NEMS-S and NEMS-R protocols<br>**Limitations:**<br>• NEMS tools need adaption for Australian context;<br>• Small sample size due to rural geography which limited statistical analysis;<br>• 'Outshopping' not accounted for;<br>• No comparison group<br>**Recommendations:**<br>• The current, predominately unhealthy, food environment provides scope to work with food retailers and consumers to ensure healthier options are more visible, available and affordable
**DuBreck et al. (2018)[52]**	To determine how junk food opportunity density in a school zone varies by neighbourhood level	Yes<br>City of London planning	Yes,<br>Glanz et al 2005	Cross-sectional; Observational	Overall results suggest urbanicity and neighbourhood socioeconomic distress are associated with children's' exposure to junk food.	**Strengths:**<br>• Non-traditional food outlets included;<br>• Inter-rater reliability high<br>**Weaknesses:**<br>• Only reviewed menus, not what's being ordered from the menus;

	of socioeconomic distress and level of urbanicity; To determine how the quality of restaurant children's menus varies by neighbourhood level of socioeconomic distress and level of urbanicity				Menus targeted to children were unhealthy regardless of rurality.	• Did not include other environments beyond restaurants<br>**Recommendations:**<br>• Urban planners and zoning officials can use these results to facilitate discussions between stakeholder groups regarding new zoning policies and programs that discourage the location of a junk food opportunity within 800m of an elementary school
**Larson et al. (2017)[44]**	A pilot of an assessment instrument to identify the types and variety of quality foods available in the corner stores in a 'food desert'.	No	No	Cross-sectional; Observational	Availability of fresh produce low, especially fresh vegetables, with little promotion; stores more likely to have canned or frozen than fresh fruit and vegetables; majority of stores provided processed meats	**Strengths:**<br>• Selection of stores representative of area; Pre-piloted survey<br>**Limitations:**<br>• Seasonal variation not accounted for as only done once (autumn);<br>• Store managers not interviewed<br>**Recommendations:**<br>• Include interviews with store managers for views on challenges in providing healthier options
**^Palermo et al. (2016)[37]**	To monitor the cost and affordability of a nutritious diet and to assess the influence of distance from the capital city and socio-economic status on the cost of nutritious food in Victoria	No	No	Longitudinal; Observational; Cohort	Fruit and vegetables contribute a large proportion of the costs of the basket and are more variable in price over time. The distance the store is from Melbourne makes the largest contribution to the variance in cost. The further the store is away from Melbourne the higher the cost. Socio-economic indices of the local government in which the store was located were not a strong predictor of VHFB cost. Average cost of the VHFB increased 6% or approximately $20 over the study period.	**Strengths:**<br>• Stores stratified based on existing data showing variation in cost based on store type (chain or independent); stratified sample selected into cohort for observation<br>• All data collectors trained in methodology<br>**Limitations:**<br>• Local governments not randomly selected, only stores<br>• Listing of supermarkets and general stores obtained from secondary sources, no primary verification<br>• Data collection carried out over 4x8 week periods, may have resulted in seasonal variation in price and quality of food<br>• Data only monitored over 3 year period, difficult to identify trends in such a short period<br>• No explanation as to why distance from capital city chosen over ARIA+<br>• Distance from capital city by road<br>**Recommendations**<br>• Need for a national approach to monitor price of what people are actually eating and of an ideal diet
**Byker Shanks et al. (2015)[45]**	To assess the consumer food environment in rural areas in Montana using NEMS-S to measure the availability, price and quality of fruits and vegetables	Yes USDA rural-urban continuum codes (RUCCs)	Yes, Glanz et al 2005	Cross-sectional; Observational	NEMS-S total scores, availability scores and price scores did not differ by county rurality; stores in the least rural counties had highest quality scores; most stores accepted the SNAP program	**Strengths:**<br>• Selection of stores using RUCC<br>• Use of validated NEMS-S tool<br>**Limitations:**<br>• Limited number of stores within a certain radius in rural counties;<br>• Caution regarding application of study findings to other localities;<br>• Long collection period of 11months due to geographical location of stores may have affected data (seasonality);<br>• 'Outshopping' not accounted for<br>**Recommendations:**<br>• Future research needed to focus on solutions to improve quality of fruits and vegetables targeting limited infrastructure for food distribution (roads, storage, frequency of delivery) in rural areas
**Byker Shanks, Jilcott Pitts & Gustafson (2015)[46]**	To develop an audit tool to measure the food environment at farmers'	Yes USDA rural-urban	Yes, Glanz et al 2005	Observational; Cross-Sectional	F-MAT can credibly be used within farmers' market sites by trained data collectors	**Strengths**<br>• Excellent rationale for tool development<br>• Describes the scale used to quantify Kappa values

	markets and to examine its psychometric properties, including face validity, discriminant validity and interrater reliability	continuum codes (RUCCS)				• Detailed discussion of statistical methods<br>• Very detailed description of how farmer's markets were chosen to pilot the tool<br>• Detailed information about reliability and validity provided and analysis included<br>• Written guidelines given to each data collector in an effort to improve inter-rater reliability<br>• Discusses limitations of study: only 3 states and only Summer months<br>• Discusses generalisability of tool (but not internationally)<br>**Limitations**<br>• Only tested in three states in the USA, unknown if applicable internationally<br>• Only piloted in Summer months (seasonal variation)<br>• No discussion about socio-economic characteristics of selected towns<br>• No funding statement<br>**Recommendations:**<br>• The F-MAT is a reliable and valid method to assess food availability and quality at farmers' markets
**^Chapman et al. (2014)[38]**	To compare food costs and variety of fresh fruits and vegetables available at three time points in December 2006, December 2008 and July 2009.	Yes,<br>ARIA (Accessibility/Remoteness Index of Australia)	No	Longitudinal; Observational; Cohort	Total food costs, and fruit and vegetable costs, were more expensive in remote areas compared with highly accessible areas; The cost of fruits and vegetables decreased over the study period by 27.4% between December 2006 and July 2009; There was no association between food costs and SES	**Strengths:**<br>• Details rationale for study<br>• Clearly states objectives<br>• Detail about remoteness, type of store and SES of area provided<br>• Good discussion of results and generalisability in relation to other Australian states and previous studies<br>**Limitations:**<br>• Food basket items based on the 2003 Dietary Guidelines<br>• Convenience sample of stores<br>• Volunteers used to collect data<br>• Variety only examined for fresh fruit and vegetables, although frozen varieties provide the same nutritional value<br>• Price comparison between winter and summer based on only one Winter price collection (2009) and compared to Summer price collection 3 years earlier (2006)<br>• Insufficient detail about the size of the stores surveyed or why differences in number of stores surveyed in different time periods<br>• No discussion of potential confounders or sources of bias<br>• No detail about how data collectors were trained or sample size determined<br>• No information about potential number of eligible stores across the state<br>• No mention of reliability or validity of tool<br>• Insufficient discussion of limitations<br>**Recommendations:**<br>• The results demonstrate the ongoing need for monitoring food costs
**Pereira et al. (2014)[32]**	To conduct a comprehensive assessment of the consumer food environment in a rural community	No	Yes,<br>Glanz et al 2005	Observational; Cross-sectional*	Findings were mixed as to which type of restaurant (fast-food or sit-down) had healthier practices; Grocery stores were more likely to have healthier products of better quality than convenience stores	**Strengths:**<br>• Detailed rationale for study provided<br>• Excellent detail about context of study<br>• Discussed exclusion of stores and reasons<br>• All data collectors received 2-day training in administering the NEMS tools<br>• Majority of retail food outlets in the rural community were assessed<br>• Good discussion of limitations and generalisability of findings<br>**Limitations:**<br>• No mention of potential bias

						• Data collection took place over nearly 12 months, this may have resulted in seasonal fluctuations in price and availability of fruit and vegetables influencing results<br>• Small sample size – therefore no statistical analysis conducted<br>• Identified NEMS –R Tool weakness – many restaurants do not provide nutrition information, therefore those that do (usually fast food restaurants) obtain a higher score regardless of the nutritional value of the food<br>• Identified NEMS-S weakness – does not assess product placement<br>**Recommendations:**<br>• Further research and evaluation post-intervention to determine if there is a change in the Consumer Nutrition Environment<br>• *Findings are intended to be used as a baseline in an intervention study yet to be reported on
**^Pollard et al. (2014)[22]**	To determine the impact of geographic factors on food pricing and quality in Western Australia (WA)	Yes, ARIA+	No	Observational; Cross-Sectional	The cost of food significantly increased with distance from the major cities (this was across all food groups); Price of a healthy food basket in remote and very remote areas of WA higher than metropolitan areas; Mean quality for fresh produce generally decreased with geographical distance from a metropolitan centre	**Strengths:**<br>• Total sample size (n =160) put in context of the total number of stores in WA (n=447)<br>• Detailed information about quality assessment tool used for fruit and vegetables<br>• Included generic brand items in HFAB survey (in contrast to VHFB)<br>• Survey conducted at same time period across all stores to minimise bias/ confounders<br>• All data collectors were trained<br>• Methods section details how missing prices were imputed in analysis<br>• High level of detail provided re: statistical analysis<br>• Provided response rate – 144 stores of the 160 selected<br>**Limitations:**<br>• Average time to complete the survey was 4.1 hrs<br>• No mention of reliability and validity testing of quality rating system used in this study<br>• 97 data collectors for 140 surveys – no mention about inter-rater reliability<br>• No discussion about potential confounders<br>• Insufficient discussion of study limitations<br>**Recommendations:**<br>• Food sovereignty plans developed locally as a food security response<br>• Call to government to support subsidies for rural and remote food transport<br>• More research is need to further develop the food quality instrument
**^Tseng et al. (2014)[39]**	To create a conceptually based index of obesogenicity representing neighbourhood characteristics thought to be related to individual risk for adiposity; To examine its association with BMI both cross-sectionally at baseline and longitudinally at a 3-year follow-up, among women in socio-	Yes, Australian Regional Infrastructure Development Fund Act 1999	Not according to a specific conceptual model of the food environment	Observational; Longitudinal; Cohort	Index items were more likely to be positively associated with BMI among urban participants but inversely associated with BMI among rural participants	**Strengths:**<br>• Author's acknowledge that residential environment may be less important than a person's activity space as individuals likely do not limit food acquisition to their immediate environment<br>**Limitations:**<br>• Data on store locations for food environment component only obtained from secondary sources<br>• Only fast food chains included in the analysis, likely to skew the results as there are fewer fast food chain outlets in outer regional/rural areas<br>• Does not take into account ability to access fast food from convenience stores, particularly relevant in a rural context<br>• Authors note that index may be more appropriate in urban as opposed to rural settings<br>• Looking at community food environment alone may not be enough

	economically disadvantaged neighbourhoods, both urban and rural across Victoria, Australia					• 2km buffer is an arbitrary distance<br>**Recommendations:**<br>• Further exploration of environmental characteristics that are obesogenic in rural settings is warranted
**Pitts et al. (2013)[33]**	To assess the consumer nutrition environment in rural county stores as a baseline measure;<br>To provide information to program planners to inform an intervention to improve the consumer nutrition environment	No	Yes,<br>Glanz et al 2005	Observational; Cross-sectional*	Rural stores had a lower mean NEMS-S-Rev score (ie. were less healthy) than stores in urban areas;<br>Corner stores in rural areas had more healthful options available than corner stores in urban areas	**Strengths:**<br>• Extensive review of audit tools before selecting validated audit tool, based on:<br>1) Ability to use tool in different types of traditional and non-traditional food stores; including supermarkets, corner stores and dollar stores<br>2) Assessment of availability, price and quality of items<br>3) Inclusion of canned vegetables and meats<br>4) Time required to complete audit<br>5) Ability to calculate a score for each food store assessed<br>6) Applicability to various communities<br>• Clearly describes measurement and efforts to reduce bias<br>**Limitations:**<br>• Very limited statistical analysis of results presented<br>• No information about the size of stores or characteristics of population<br>• Limited analysis of sub-groups and small sample sizes<br>• Small, non-random sample of food stores<br>**Recommendations:**<br>• Future assessments should combine measurement of the consumer nutrition environment and the community nutrition environment<br>• *Findings intended to be used as a baseline in an intervention study yet to be reported on
**^Innes-Hughes et al. (2012)[40]**	To assess the community and consumer food environment in three small rural towns in New South Wales, Australia	No	Yes,<br>Glanz et al 2005	Observational; Cross-Sectional	Considerable number of take-away food outlets in each town;<br>Supermarkets had the most consistent availability of healthy indicator foods;<br>Residents in all towns had ready access to energy-dense, nutrient-poor snack foods and beverages	**Strengths:**<br>• Trained researches collected data<br>• Ethics approval obtained<br>• Secondary data sources (yellow pages) used to identify food outlets, results verified with primary observation<br>• Reliability and validity considered<br>• Uses a conceptual model as a basis for research<br>• Looks at both the community and consumer nutrition environments<br>• Based tools on NEMS-S and NEMS-R measures<br>**Limitations:**<br>• Assumption made that tools developed do in fact measure the constructs which they are designed to test<br>• Did not consider quality<br>• Insufficient discussion of study limitations<br>• Small sample size<br>**Recommendations:**<br>• Data could be used to prompt further local data collection<br>• A more substantial dataset on food availability would be advantageous across a number of towns
**^Ward et al. (2012)[41]**	To determine if there is a difference in cost and affordability for a 'healthy food basket' between rural and	Yes,<br>ARIA	No	Observational; Cross-Sectional	Cost of healthy food basket higher in rural areas across all reference family types;	**Strengths**<br>• Authors acknowledge that 'healthy food basket' items may not reflect what people actually eat<br>**Weaknesses**

	metropolitan sites and different levels of SES				No statistically significant difference in affordability between metropolitan and rural areas; Affordability varied by SES in both rural and metropolitan areas	• Very small sample size for rural supermarkets (n=14) compared to metropolitan (n=64)<br>• Insufficient detail about how study size was determined<br>• No information about whether any eligible supermarkets refused to participate/denied entry<br>• Lacks detail about the size and type of supermarkets studied<br>• No detail about how towns for the study were selected<br>• Very limited discussion about potential confounders<br>**Recommendations:**<br>• Recommends monitoring food affordability longitudinally
**Sadler, Gilliland & Arku (2011)[53]**	To uncover deficiencies seen in rural studies on food environments and improve the methods used to characterise rural food accessibility	Yes, Organisation for Economic Cooperation and Development, Canada	No	Observational; Cross-sectional	Residents in the most distressed neighbourhoods tended to have better accessibility to all types of food retailers; average accessibility to all food retailer types improved dramatically when food outlets adjacent to the study area were considered, thereby controlling for the 'edge effect'.	**Strengths:**<br>• Methods of analysis rely on road network when calculating distances and interpreting access thresholds – specific to rural conditions<br>• Included food stores outside the geographical boundary of interest<br>• Assigned distance scores to individual address points for each residence, rather than using an aggregate. Authors state that this is important due to large swaths of rural areas being unpopulated<br>**Limitations:**<br>• No discussion of when the 'edge effect' might be more pronounced, ie. in rural areas situated close to a large town/centre. Unlikely to be so in more isolated rural areas<br>• No discussion of study limitations<br>**Recommendations:**<br>• Future researchers of rural environments to consider the methodology employed in this study
**Sharkey et al. (2011)[47]**	To ascertain the potential spatial access to healthier fast-food options;<br>To examine the relationship between neighbourhood deprivation and spatial access to all fast food opportunities and to fast-food opportunities that offer healthier options	No	No	Observational; Cross-sectional	A greater proportion of traditional food stores compared with fast-food restaurants offered healthier options in a variety of side dishes; Identifying fast-food restaurants as the sole source of fast-food entrees and side dishes underestimated neighbourhood exposure to fast food, in terms of both proximity and coverage; Median distance to the nearest fast-food opportunity was 2.7 miles, compared to 4.5 miles to the nearest fast-food restaurant; Access to fast-food in terms of proximity and coverage significantly improved across neighbourhoods of increasing deprivation	**Strengths**<br>• Appropriate statistical tests employed with sufficient detail provided<br>• Surveys non-traditional fast food outlets which gives a more detailed picture of fast-food access than simply surveying fast food restaurants<br>**Limitations:**<br>• No explanation provided as to why entrees and side dishes were the subject of the study<br>• ≥ 2 healthy options was considered a 'variety' of healthy options<br>• Used data from the 2000 US Census for GIS mapping residential centroids<br>**Recommendations:**<br>• Potential interventions must consider all retail opportunities for fast food, not just traditional fast food outlets
**Smith et al. (2010)[50]**	To determine if there is a difference in access to grocery stores selling fresh fruit and vegetables in island, rural, small town and urban Scotland	Yes, Scottish Executive's Urban-Rural Classificatio	No	Observational; Cross-sectional	Lower SES communities had better spatial access to grocery stores in urban Scotland;<br>Poor spatial accessibility to grocery stores in deprived neighbourhoods may exist in island, rural and small town settings	**Strengths:**<br>• Surveyed a geographical and SES range of sentinel sites<br>**Limitations:**<br>• Potential confounders/possible bias not well described<br>• No mention of any stores which refused entry<br>• Only considers access to fresh fruit and vegetables, not a range of healthy food items required for a nutritious diet

		n Scheme (SEUR)				• Obtained food retail outlet information for surveying and GIS mapping from secondary sources<br>• HEISB Tool includes 17 fruit and vegetable items, only 12 were included in this study<br>• No indication as to why travel times were used in analysis as opposed to geographical distance<br>• Assumption that residents patronize the nearest store to their home<br>**Recommendations:**<br>• In Scotland, policies and interventions targeted at more rural and remote communities may help reduce spatial inequalities in diet and diet-related chronic diseases
**Wang et al. (2010)**[54]	To examine the availability and accessibility of 'healthy' foods in rural and urban New Zealand	No	No	Observational; Cross-Sectional	'Healthy' food choices were more available in urban than rural areas; 'Healthy' food choices were more expensive than 'regular' choices in both rural and urban areas – this was more pronounced in urban areas	**Strengths**<br>• Large sample size (n=1230 stores)<br>• Surveyed all stores in 12 geographical areas<br>• Ethics approval obtained<br>**Weaknesses/Limitations:**<br>• Insufficient detail about how the 'healthy food basket' of 15 items was determined<br>• No detail about the criteria for determining 'healthy' options in restaurants and takeaway food stores<br>• Only outlets which stocked both 'regular' and 'healthy' alternatives were included in analysis for price comparison – likely to skew results<br>• Comparison of results between urban and rural areas does not take into account the difference in sample size in each area<br>• Fruit, vegetables and fish not included in price comparisons as there are no 'unhealthy' comparison items<br>• No detail about whether data collectors trained in instrument use<br>• No psychometric testing of the tools developed or detail about training of data collectors<br>• Insufficient discussion about limitations of assessment methodology<br>**Recommendations:**<br>• Improvement in the food environment is needed to facilitate the adoption of healthy food choices
**Cummins et al. (2009)**[51]	To determine if produce quality systematically varies by food store type, rural-urban location and neighbourhood deprivation in a selection of communities across Scotland	Yes, Scottish Executive's Urban-Rural Classificatio n Scheme (SEUR)	No	Observational; Cross-sectional	Overall the quality of fruit and vegetables within the surveyed stores was high;<br>In all cases, stores where food is secondary had the lowest quality scores or did not stock particular items;<br>Although not statistically significant, poorer quality fruit and vegetables were found in urban stores, stores in deprived neighbourhoods and in stores where food was a secondary product	**Strengths:**<br>• Relatively large sample of stores surveyed<br>**Limitations:**<br>• Possible confounders/sources of bias not discussed<br>• Uses only secondary data sources to locate retail food outlets<br>• Data collected in two phases: October/November 2005 and February/March 2006 which may have influenced the results based on seasonal variability in fresh produce quality<br>• Did not investigate inter-rater reliability<br>• The 3 point Likert scale may not have been sensitive enough to capture the full range of variation in quality of the produce<br>• Cross-sectional design does not account for seasonal variation in quality<br>• No information about whether any stores refused data collector entry<br>**Recommendations:**<br>• Data suggest that the quality of fruit and vegetable items may be worth investigating as a plausible micro-environmental determinant of purchasing and consumption behaviour

**^Palermo et al. (2008)**[42]	To investigate the factors that influence cost of food in rural and regional Victoria	Yes, ARIA	No	Observational; Cross-Sectional	The cost of healthy food in rural Victoria varies in a manner that appears unrelated to remoteness, population, SES or distance from Melbourne;<br>Median cost of VHFB for a 'typical' family was significantly higher at independent grocers than chain stores;<br>No association between SES and food cost;<br>Greater variation in cost of healthy compared to unhealthy foods	**Strengths:**<br>• Healthy food basket developed using nutrient reference values for a number of different family types<br>**Limitations:**<br>• Possible confounders and sources of bias not discussed<br>• Explains that sample was convenience sample but no explanation of how study size determined<br>• No mention of if any stores refused entry and how this impacted on sample size<br>• No mention of whether student data collectors were trained in the use of the instrument<br>• Inner regional, outer regional and rural towns all considered as a homogenised group<br>• 40% of the 20 largest Victorian rural and regional towns were included in the sample, but less than 5% of the smaller rural and regional towns included<br>• VHFB does not include generic items<br>• Only 2 'unhealthy foods' included in the sample – difficult to make inferences about price variations<br>**Recommendations:**<br>• National food price monitoring system required
**Hosler et al. (2008)**[48]	To investigate and compare the availability of fruit and vegetables in urban and rural underserved communities in New York State	Yes, US Census Bureau	No	Observational; Cross-sectional	The urban minority neighbourhood had the most barriers to accessing fresh fruit and vegetables in retail outlets, attributed to the lack of supermarkets	**Strengths:**<br>• Included all food stores in the study communities<br>• Thorough process for identifying potential stores for inclusion<br>• Included non-traditional sources of fruit and vegetables (road side stalls and farmers markets) – even though these were not well defined<br>**Limitations:**<br>• Surveyed stores from July – September 2003 – long period and introduction of potential confounders<br>• Insufficient description of how the settings for investigation were selected and SES status of each area<br>• Stores only needed to stock one item of bread, milk or fruit and vegetables to be included in the study, however, study objective is about fruit and vegetable availability<br>• Only tested inter-rater reliability - not validity, specificity or test-re-test reliability<br>• Originally used standard store classification system and then decided to develop own system based on results<br>• Based weighted calculations for density on numerous untested assumptions using a complicated equation not supported by previous literature<br>• Does not take into account where the stores are geographically situated in relation to population<br>**Recommendations:**<br>• Simple methods to measure food access need to be developed which can be used in urban and rural areas
**Creel et al. (2008)**[49]	To identify opportunities for the procurement of fast-food in a rural region of Texas, USA;<br>To determine the extent to which a variety of	No	No	Observational; Cross-sectional	53.2% convenience stores, 41% fast food outlets, and 5.8% supermarkets;<br>Convenience stores offered less variety in healthier breakfast and lunch/dinner options than either	**Strengths:**<br>• Inclusion of non-traditional outlets in the sample<br>• Classified stores according to the North American Industry Classification System<br>**Limitations:**

	regular and healthier fast-food options are associated with the type of business (ie. traditional fast food outlet, convenience store, supermarket/grocery)				tradition or fast-food outlets or supermarkets;<br>Convenience stores and supermarkets provided more than double the potential availability of fast food than traditional fast food restaurants alone	• What constituted 'fast food' was insufficiently described as an inclusion criteria<br>• No discussion of potential confounders<br>• Insufficient detail regarding how sample size was determined<br>• No information about total number of stores sample drawn from or whether any stores refused entry<br>• Insufficient descriptive data about store location (ie. SES)<br>• No consideration of validity or reliability of tools developed<br>• No use of theoretical framework to conceptualise food environment<br>• No link to actual tool for review<br>**Recommendations:**<br>• Convenience stores should be targeted for expansion of healthier food offerings<br>• Convenience stores and supermarket/ grocery stores more than double the potential access to fast foods in this rural area when compared to traditional fast-food outlets alone
**^Herzfeld & McManus (2007)[43]**	To develop and trial a survey tool that could be used to gather evidence relating to vegetable and fruit availability, quality and price in urban fringe and rural communities	Yes,<br>ARIA	No	Cross-Sectional;<br>Observational	The pilot tool was time consuming to administer and demonstrated poor inter-rater reliability	**Strengths:**<br>• Provides an operational definition of 'availability'<br>• Data collectors gathered additional data about produce quality including date of delivery, storage conditions and, source of produce.<br>• Considers validity (but did not test it)<br>• Tests inter-rater reliability<br>• Undertook literature review to determine what criteria to use in the survey tool<br>**Limitations:**<br>• Insufficient detail provided about background and rationale for study<br>• Criteria used to determine items included in the survey not based on dietary guidelines or a reflection of consumption patterns<br>• No link provided to the tool for review<br>• No information about potential confounders or possible bias<br>• Very poor inter-rater reliability for quality<br>• Very poor inter-rater reliability for price<br>**Recommendations:**<br>• Further work to simplify the format of the survey and ensure ease of use; and strengthen validity and reliability<br>• Data collectors could receive training 'in situ' to ensure standardisation of methods
**Martínez-Donate et al. (2015)[31]**	To pilot test a community level intervention to improve the nutrition environment and promote healthy eating in restaurants and food stores in a rural community	No	Yes,<br>Glanz et al 2005	Quasi-experimental	Intervention site demonstrated increase in average NEMS-R score in restaurants; The NEMS –R scores improved due to: healthy signage, identification and promotion of healthier foods; No changes in NEMS-S scores in supermarkets	**Strengths:**<br>• Towns selected which did not have other healthy eating initiatives to minimise confounding results<br>• Based interventions on the social ecological model of health theory<br>• Intervention site and a control<br>• Ethics approval obtained<br>• Post-intervention data collected by blinded assessor<br>• Paired t-tests used to determine intervention effects<br>**Limitations:**<br>• Relatively short intervention period (10 months)<br>• Insufficient detail about the timing of the intervention and how much support was provided to stores/restaurants<br>• Study objectives not specifically stated early in the paper

						• Insufficient detail about how communities were assigned to intervention or control and how bias was minimised<br>• Multiple threats to validity with only one intervention and one control site<br>• Poorly matched intervention and control communities in terms of population size<br>• Sample did not include any convenience stores (the one convenience store approached declined to participate)<br>**Recommendations:**<br>• Intervention should be interpreted cautiously due to small sample size, however, it seems the environment in restaurants was more successfully altered and maintained than supermarkets<br>• Needs to be remembered that this study was not designed to test effectiveness, but to demonstrate feasibility and acceptability
